# Novel Anti Co-Channel Interference Scheme for Sensor Networks

**DOI:** 10.3390/s100403170

**Published:** 2010-03-31

**Authors:** Man Feng, Lenan Wu

**Affiliations:** Institute of Information Science and Engineering, Southeast University, Nanjing 210096, China; E-Mail: wuln@seu.edu.cn

**Keywords:** interference suppression, wireless sensors network, Extended Binary Phase Shift Keying (EBPSK), filter

## Abstract

With improvement of the automation level, wireless sensors are widely used, but various kinds of interference lead to problems in the application. In order to deal with co-channel interference, a throughput efficient scheme based on Extended Binary Phase Shift Keying (EBPSK) modulation is introduced in physical layer. On this basis, the corresponding transmission scheme and the important impacting filter are presented. Effects of co-channel interference on the EBPSK waveform in different cases are analyzed. Simulation results illustrate the excellent anti-interference performance of the EBPSK system itself, when the initial phase of the co-channel interference is small.

## 1. Introduction

With recent developments in modern science technology, various types of sensors have been widely used in industry, agriculture, aerospace, aviation and other fields, to allow the production process a high level of automation. However, the interference suppression in the application of wireless sensors is a very difficult problem, which affects the control precision and accuracy of the whole system. Therefore, a variety of anti-interference measures to improve system stability and accuracy are inevitable, which has been a subject of intense research. The usual methods include shielding technology, filtering technology, isolation measure and grounding technology, *etc.* In some special cases, even more complicated anti-interference methods [1–4] are needed, but the performances are still not satisfactory, for example, Reference [1] proposed an interference avoidance frequency assignment method IAFA, Reference [2] studied explicit definitions of interference and proposed various models of interference to ensure the reasonable anti-interference methods, Reference [3] used the second order vibration mode to solve the co-channel interference in the silicon resonant beam microsensor, Reference [4] proposed a novel measure with nonsymmetrical excitation to eliminate the co-channel interference, *etc.*

This paper considers this problem in physical layer directly in order to remove the complicated anti-interference module. In our research, we have found that the Ultra Narrow Band (UNB) system [5,6] based on Extended Binary Phase Shift Keying (EBPSK) [6] modulation presents good performance of anti-interference, which may have very wide applications, and can realize high data rate transmission within an ultra narrow bandwidth. On the other hand, its modulated waveforms corresponding to “0” and “1” have a very tiny difference, if demodulating by using classical correlation detector or matched filter, it has even high demand on input SNR. In order to improve the efficiency of the EBPSK signal as much as possible, the special detection filter method [7,8] must be sought, which can amplify the signal characters as much as possible and remove utmost noise. However, traditional filter theory and conventional filter [9] design can hardly satisfy this demand. For example, after the EBPSK modulation signal is filtered by narrow classical Infinite Impulse Response (IIR) or Finite Impulse Response (FIR) digital filters, the carried information is also removed, and just pure sine wave as carrier remains in the filter center frequency. [8] proposed the impacting filter based on zero-pole point, which can cooperate with tiny waveform difference of EBPSK signal to transform the phase jumping information into amplitude impacting. Because of the special characteristics of the EBPSK modulation waveform, the co-channel interference can be thought of as useful information to add in the modulation waveform, and have no effects on the BER performance, *i.e.,* EBPSK system itself has the better performance in canceling co-channel interference.

All in all, aiming at the interference problem in wireless sensors networks, this paper proposes a simple solving scheme of physical layer, which is based on EBPSK modulation with ultra narrow bandwidth. Simultaneously, the anti-interference performance of the EBPSK system itself is mainly discussed. When the initial phase of the co-channel interference is small, the EBPSK system has good anti-interference characteristics, which has been analyzed by intuition and proved by simulation results. While with the increasing of the initial phase, the anti-interference performance of system itself decreases, so the usual anti-interference methods can be considered to solve it.

The paper is organized as follows. The EBPSK modulation and the analysis of the related parameters are introduced in Section 2. Section 3 presents the scheme of EBPSK transmission system. The effects of co-channel interference are analyzed and verified experimentally in Section 4. Then BER simulation results are given in Section 5. Finally, Section 6 gives some conclusive remarks.

## 2. EBPSK Modulation and Transmission System

EBPSK [6] is a modulation method with high frequency spectra efficiency, which is defined as following:

(1) $$\begin{array}{c} g_{0}(t)=A\;\text{cos}\;2\pi f_{c}t,0\leq t<T\\ g_{1}(t)=\{\begin{array}{cc} B\;\text{cos}\left(2\pi f_{c}t+\theta \right), & 0\leq t<\tau ,0\leq \theta \leq \pi \\ A\;\text{cos}\;2\pi f_{c}t, & \tau \leq t<T \end{array} \end{array}$$

where *g*_0_ (*t*) and *g*_1_ (*t*) represents the waveform modulated by “0” or “1”, respectively, *T* = 2*π N/ω_c_* = *NT_c_* is the symbol width of data, *T_c_* = 2*π*/*ω_c_* = 1/*f_c_* is the carrier cycle, namely *T* lasts *N* ≥ 1 cycles of carrier; *T* is the symbol width of data; *θ* is the phase jumping angle or modulating angle, and *τ* is the time length of the *θ* jumping lasting. The paper just considers the case of *A* = *B* = 1.

In EBPSK modulation, the modulated waveforms corresponding to “0” and “1” have a very tiny difference, and the most parts of the waveform are sine components, therefore, having compact power spectra [10], *i.e.,* high throughput efficiency, and is difficult to detect. The problem faced is how to design a UNB receiver filter to save or improve phase jumping information. The traditional IIR or FIR filter can erase the phase information, so that “0” and “1” information cannot be distinguished. A special UNB impacting filter [8] will be used in EBPSK system, which can transform the tiny phase information into the higher impulse amplitude at the phase jumping point corresponding to code “1” than the amplitude of “0” [11], that is to say, amplify the signal characters as much as possible and remove the maximum noise and other interferences. Consequently, in the case of precise synchronization, we can use directly amplitude detection at the jumping point [12] or energy accumulation of tailing to judge “0” and “1”, which causes the structure of receiver much simplifier than that based on phase locked loop [6]. Therefore, the UNB transmission system can be digitally implemented, since the EBPSK has covered both the traditional BPSK and most UNB modulations, which is beneficial for chip integration.

## 3. Analysis of Effects of Co-channel Interference on EBPSK Waveform

**Figure 1.**
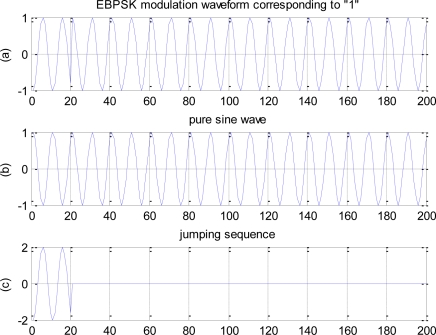
The decompose of the EBPSK signal. (a) EBPSK signal. (b) Pure sine wave. (c) Jumping sequence.

**Figure 2.**
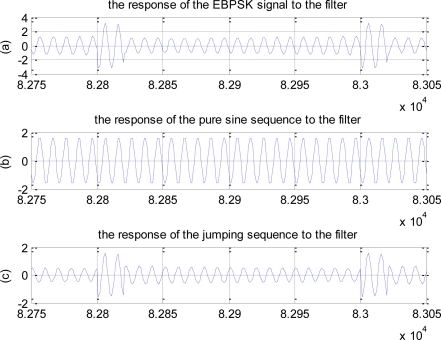
The response of the decomposed sequence to the filter. (a) The response of the EBPSK signal to the filter. (b) The response of the pure sine sequence to the filter. (c) The response of the jumping sequence to the filter.

The modulation waveform corresponding to code “1” can be regarded as the superposition of all-zero sequence (*i.e.,* pure sine wave) and jumping sequence (*i.e.,* just existing signal in EBPSK jumping point), as shown in Figure 1. In addition, the special detection filter used in EBPSK system is a linear filter, so superposition principle can be used. For simplicity, this paper takes the filter having one conjugate pole and one conjugate zero points as an example to analyze. Letting the decomposed sequences pass the filter, we can obtain the response shown in Figure 2. Obviously, the jumping response and all-zero response have almost the same phases, which enhance each other, so that the amplitude impulse is produced.

Reference [8] proposed the IIR narrowband band-pass filter with multi-poles and one zero, which can produce even much higher impact in phase jumping point than that with one pole and one zero as analyzed above, much narrower bandwidth, and greatly improve output SNR. Especially, in the case of signal submerged by noise, *i.e.,* SNR < 0, the modulation information is emphasized with impacting shape, therefore, in the following real simulation, the filter having three conjugate poles and one conjugate zero point is taken as an example, and the transfer function can be written as following:

(2) $$H(z)=\frac{1-1.61817331\;\;\;85991785\;z^{-1}+z^{-2}}{1+\sum_{i=1}^{6}a_{i}\cdot z^{-i}}$$

where *a*_1_ = −4.5781931992746454, *a*_2_ = 9.6546659241157258, *a*_3_ = −11.692079 480819313, *a*_4_ = 8.5756341567768217, *a*_5_ = −3.6121554794765309, *a*_6_ = 0.70084076 00731199

### 3.1. Effects of Interferences with Different Initial Phase on Time-Domain Waveform

**Figure 3.**
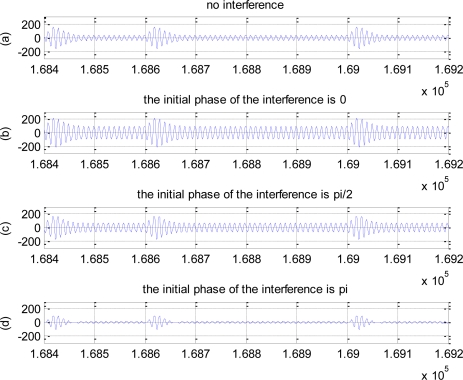
The effect of different initial phase on impulse amplitude. (a) No interference. (b) The initial phase of the interference is 0. (c) The initial phase of the interference is *π*/2 (d) The initial phase of the interference is *π*.

Considering co-channel interference, if its initial phase is 0, the added interference can be thought of as the amplitude increasing of all-zero sequence in the decomposed sequences, *i.e.,* in-phase stacking, which will cause both the amplitudes of jumping part and non-jumping part to increase, but the amplitude difference remains unchanged. On the other hand, with the increasing of the initial phase, the phase difference of the interference response and all-zero response become larger, and the amplitude enhancement role becomes poor, *i.e.,* the part of waveform will be cancelled because of the existing of the phase difference, thus, the impulse amplitude decreases too. Figure 3 gives the time-domain response waveforms in no interference, and when initial phase is 0, *π*/2 and *π*, respectively, where *SIR* = 0*dB*, which verifies the correctness of the above intuition explanation. All in all, the produced impulse in the case of the zero initial phase is much higher than no interfered and other initial phase cases.

### 3.2. Effects of Different Signal to Interference Ratio (SIR) on Time-Domain Waveform

**Figure 4.**
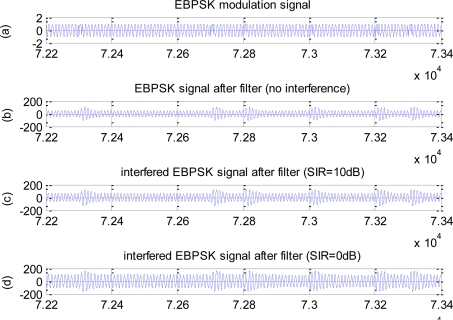
Effects of different SIR on time-domain waveform. (a) EBPSK modulation signal. (b) EBPSK signal after filter (no interference). (c) Interfered EBPSK signal after filter (*SIR* = 10*dB*). (d) Interfered EBPSK signal after filter (*SIR* = 0*dB*).

When signal power remains unchanged, the amplitude of the interference decreases with the increasing of SIR, *i.e.,* the energy of the corresponding sine is weakened, thus, the enhancement role for signal becomes poor according to section 3.1. Therefore, the impulse amplitude decreases, but the amplitude difference remains unchanged. Figure 4 gives the response to filters in the time-domain of no interference, *SIR* = 0*dB* and *SIR* = 10*dB*, respectively, where the initial phase of the interference is 0.

## 4. BER Analysis

In this section, computer simulation results of bit error rate (BER) are given to illustrate the good anti-interference characteristics of the EBPSK system, where the transfer function of the filter is chosen as (2), *i.e.,* simulations are based on the filter having three conjugate poles and one conjugate zero. On the other hand, just co-channel interference is considered in this paper. In our simulation, the other parameters are chosen as follows: *f_c_* = 930*kHz*, *N* = 20, *K* = 2, *A* = *B* =1, *θ* = *π*.

### 4.1. Effects of Different SIR on BER Performance

**Figure 5.**
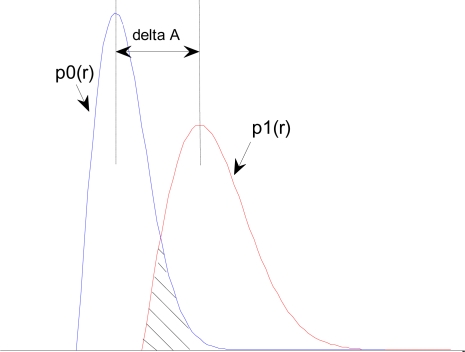
The sketch map of the detection performance by pdf.

First, we consider BER performances in different SIR cases. Outwardly, according to Section 3.2, the impulse amplitude of jumping part increases with the decrease of SIR, but simultaneously the amplitude of non-jumping part corresponding to “0” is also improved, *i.e.,* the relative amplitude differences remain unchanged in different SIR cases. Theoretically, the responses of code “0” and “1”signal to filter are with Rice amplitude distributions, whose probability density function (pdf) is *p*_0_ (*r*) and *p*_1_ (*r*), respectively, just having different mean values, *i.e.,* existing the amplitude difference Δ*A*, so the BER formula of EBPSK has been deduced based on the classic detection theory [13]. Based on the binary detection model, the sketch map of the detection performance illustrated by pdf is shown in Figure 5, where the shaded area indicates the BER. In co-channel interference and small initial phase cases, the basic amplitude *A*_0_ increases, *i.e.,* both the two pdfs move to the right, but the amplitude difference Δ*A* remains unchanged, *i.e.,* the relative location remains unchanged so that the overlapped area between the two pdfs is also unchanged, therefore, the BER performance does not change in different SIR cases by present amplitude detection method. On the other hand, in the large initial phase cases, the amplitude difference Δ*A* decreases, *i.e.,* the distance of *p*_0_ (*r*) and *p*_1_ (*r*) is closer, so the overlapped area becomes large, *i.e.,* the BER increases.

**Figure 6.**
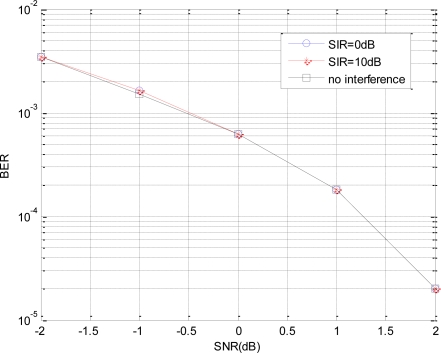
BER performances in different SIR.

Figure 6 shows the BER performances in different SIR cases, where the initial phase of the interference is assumed as 0. Obviously, the BERs are almost identical in *SIR* = 0*dB*, *SIR* = 10*dB* and no interference, which agrees with our above BER analysis. Different to the normal cases, the BER performance of the EBPSK system does not decrease when the co-channel interference is existing, which implies good anti-interference performance of this high-efficiency EBPSK transmission system.

### 4.2. Effects of Different Initial Phases on BER Performance

**Figure 7.**
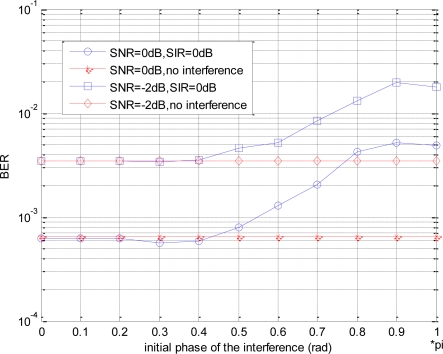
Effects of different initial phases on BER performance.

In this section, the effects of the co-channel interference with different initial phases on BER performance are simulated. Figure 7 shows the BER performances in different interference initial phases, where assuming *SNR* = 0*dB* and *SIR* = −2*dB*, respectively.

Obviously, in both cases, BER curves have almost the same change tendency with the increasing of the initial phase. When the initial phase of the interference is from 0 to 0.4*π*, the BER agrees with the case of no interference basically. However, when the initial phase is larger than 0.4*π*, the BER becomes poor with the increasing of the initial phase.

When the initial phase of the interference is small, the interference plays the enhancement role for the signal, analyzing as Section 4.1, so the BER performances are similar in the different SIR cases. On the other hand, when the initial phase becomes larger, the interference has negative effect on the original signal, especially greater on the amplitude of the jumping part, so that the waveform amplitude after filtering decreases greatly, and the amplitude difference between jumping part and non-jumping part decreases thereupon, also as shown in Figure 3. Thus, when using the threshold detection in the receiver, the larger the initial phase is, the poorer the BER performance will be.

**Figure 8.**
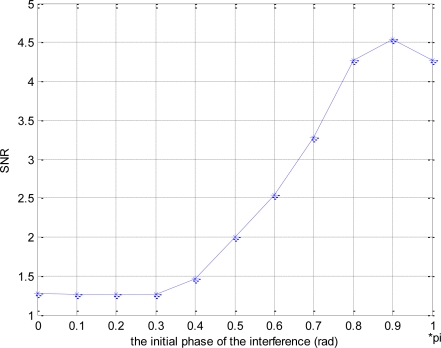
The SNR loss produced by co-channel interference.

Figure 8 shows the SNR loss produced by co-channel interference, *i.e.,* in order to obtain the same BER, such as 10^−4^, the required SNR is given for the different initial phase, which indicates the effects of the initial phase on BER more clearly.

## 5. Conclusions

1. In this paper, aiming at the interference problem in wireless sensor networks, a simple solving scheme of physical layer is proposed, which is based on EBPSK modulation.
2. EBPSK modulation as a new idea, which takes some current binary modulations as its special examples, has good flexibility, universality and good possibilities for new high speed and high efficiency transmission [10].
3. The special impacting filter can transform the tiny waveform difference into amplitude impacting, so that the receiver structure can be simplified by threshold detection [7,8]. The EBPSK transmission system can be digitally implemented which is benefit for chip integration and advances the EBPSK scheme to practicality.
4. This paper discusses the anti-interference performance of EBPSK system itself. When the initial phase of the co-channel interference is small, EBPSK system has good anti-interference characteristics, which has been analyzed by intuition and proved by simulation results. While with the increasing of the initial phase, the anti-interference performance of system itself decreases, so the usual anti-interference methods can be considered to solve it. Therefore, the anti-interference performance may extend the use of EBPSK system in interference environments. Researches on this problem are underway, so for the case of the large interference initial phase, the detailed anti-interference scheme based on the EBPSK system would be given in the forthcoming papers.
